# Uncoupling Neogenin association with lipid rafts promotes neuronal survival and functional recovery after stroke

**DOI:** 10.1038/cddis.2015.109

**Published:** 2015-05-07

**Authors:** A P Shabanzadeh, N G Tassew, K Szydlowska, M Tymianski, P Banerjee, R J Vigouroux, J H Eubanks, L Huang, M Geraerts, P D Koeberle, B K Mueller, P P Monnier

**Affiliations:** 1 Toronto Western Research Institute, Genetics and Development Division, KDT 8-418, 60 Leonard Street, Toronto, M5T 2S8 ON, Canada; 2 Department of Anatomy, Faculty of Medicine, University of Toronto, Toronto, ON, Canada; 3 Department of Physiology, Faculty of Medicine, University of Toronto, Toronto, ON, Canada; 4 Department of Surgery, Faculty of Medicine, University of Toronto, Toronto, ON, Canada; 5 AbbVie Bioresearch Center, 100 Research Drive, Worcester, MA 01605, USA; 6 Neuroscience Research, Research and Development, AbbVie Deutschland GmbH & CO KG, Knollstrasse, D-67061 Ludwigshafen, Germany; 7 Department of Ophthalmology, Faculty of Medicine, University of Toronto, Toronto, ON, Canada

## Abstract

The dependence receptor Neogenin and its ligand, the repulsive guidance molecule a (RGMa), regulate apoptosis and axonal growth in the developing and the adult central nervous system (CNS). Here, we show that this pathway has also a critical role in neuronal death following stroke, and that providing RGMa to neurons blocks Neogenin-induced death. Interestingly, the Neogenin pro-death function following ischemic insult depends on Neogenin association with lipid rafts. Thus, a peptide that prevents Neogenin association with lipid rafts increased neuronal survival in several *in vitro* stroke models. In rats, a pro-survival effect was also observed in a model of ocular ischemia, as well as after middle cerebral artery occlusion (MCAO). Treatments that prevented Neogenin association with lipid rafts improved neuronal survival and the complexity of the neuronal network following occlusion of the middle artery. Toward the development of a treatment for stroke, we developed a human anti-RGMa antibody that also prevents Neogenin association with lipid rafts. We show that this antibody also protected CNS tissue from ischemic damage and that its application resulted in a significant functional improvement even when administrated 6 h after artery occlusion. Thus, our results draw attention to the role of Neogenin and lipid rafts as potential targets following stroke.

Ischemic stroke is of major public health significance as it may lead to permanent loss-of-functions or death. This is due to the pronounced susceptibility of adult central nervous system (CNS) neurons to undergo apoptotic death when injured. Many clinical trials have focused on reducing excitotoxicity to ameliorate neuronal death in the penumbra.^1^ However, the short duration of excitotoxicity following stroke does not allow for effective treatment in the clinic. There is emerging consensus that a better therapy should be obtained by (i) targeting the molecular mechanisms of apoptosis and (ii) using this knowledge to develop effective treatments that maintain adequate brain functions.^2^

The transmembrane protein Neogenin is a dependence receptor that causes death or survival depending on ligand (repulsive guidance molecule a (RGMa)) absence or presence, respectively.^3, 4^ In cell cultures, as well as in the developing chick brain, Neogenin induces apoptosis in the absence of RGMa.^3^ Cell survival can be rescued either by addition of RGMa or by Neogenin silencing. We recently have demonstrated that RGMa can also rescue neuronal cell death following traumatic CNS injury.^5^ When retinal ganglion cell (RGC) axons were severed by optic nerve crush, injection of RGMa into the vitreous significantly increased cell survival.^5^ Thus, the Neogenin/RGMa pathway is involved in neuronal cell death following injury. More recent studies revealed that this pathway is involved in axonal regeneration following stroke. RGMa is upregulated in the penumbra of human patients who died of stroke.^6^ Interestingly, electrical stimulation downregulates RGMa expression, which correlates with an improved functional outcome following middle cerebral artery occlusion (MCAO).^7, 8^ Although Neogenin has been shown to be expressed in the injured brain following stroke,^9^ there is no direct evidence that it may have a role in the pathology of this disease.

The plasma membrane of cells contains a combination of glycosphingolipids and protein receptors organized in glycolipoprotein microdomains, termed lipid rafts.^10^ One key difference between lipid rafts and the plasma membranes from which they are derived is lipid composition. Lipid rafts generally contain twice the amount of cholesterol than that found in the surrounding bilayer.^10^ We recently discovered that RGMa contains three sites of interaction with Neogenin.^11^ Two of these sites interact with Neogenin to block axonal growth, whereas the third site, located in the most N-terminal portion of RGMa (N-Raft), binds the Neogenin immunoglobulin domain (4Ig), to regulate recruitment of Neogenin into lipid rafts. Treatment with either 4Ig or a newly generated monoclonal antibody (mAb) abolished Neogenin-induced cell death suggesting that Neogenin recruitment into rafts is essential for Neogenin-mediated apoptosis. In this study, we assessed the neuroprotective effects of RGMa, as well as, the effect of altering Neogenin association with lipid rafts after cerebral– and retinal–ischemic injuries.

## Results

### RGMa promotes RGC survival following ischemia

**Figure 1:**
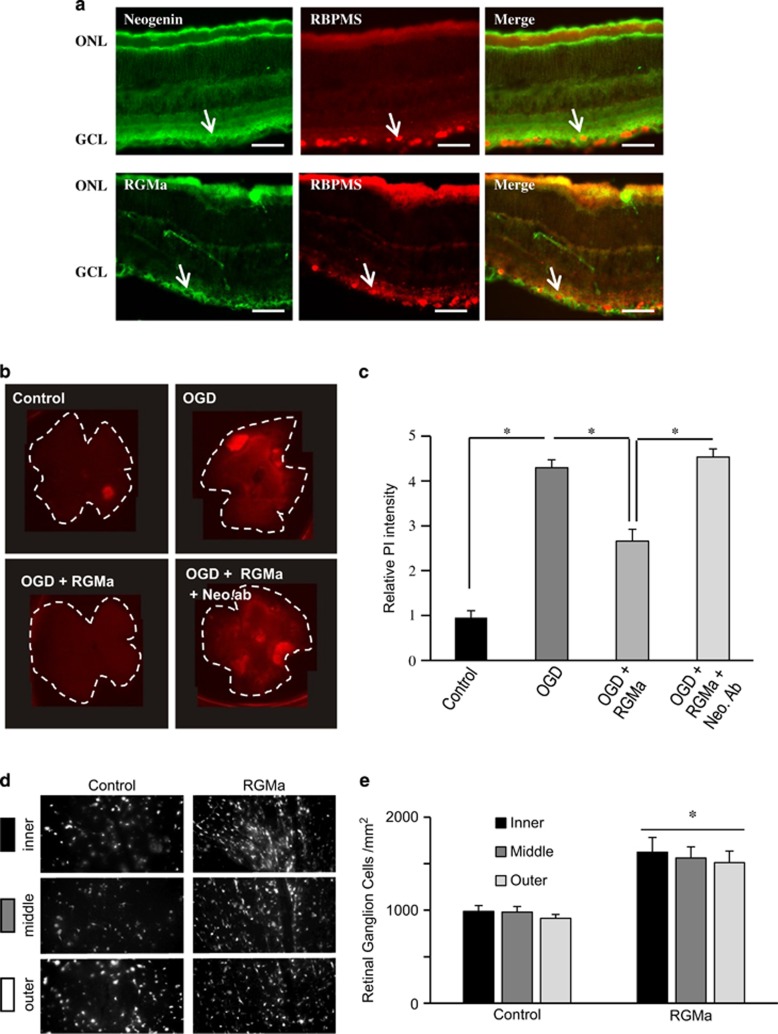
RGMa promotes RGC survival after ischemic insult. (**a**) Rat retina sections were stained with antibodies for Neogenin and RGMa and a marker for ganglion cells (RBPMS). Arrows indicate ganglion cells that express Neogenin or RGMa. ONL, optic nuclear layer; GCL, ganglion cell layer. Bar, 100 *μ*m. (**b**) Retinal whole mounts were put in culture and submitted to OGD (1 h). One day after OGD, whole mounts were stained with PI to assess cellular death. Addition of RGMa to the medium reduced the OGD-induced cellular death. A Neogenin-neutralizing antibody (Neo-ab) suppressed the pro-survival effect of RGMa. (**c**) Quantifications of experiments presented in **b** show that RGMa significantly improved cell survival, an effect abolished by addition of anti-Neogenin (*P<0.005). (**d** and **e**) Rat eyes were submitted to ischemic insult (ligation of the optic artery) and injected with BSA (control) or RGMa. (**d**) Fluorescence micrographs of flat-mounted retinas showing Fluorogold-labeled RGCs at 14 days following ophthalmic artery ligation and treatments. Representative confocal micrographs taken in the inner, middle and outer retina. Control retinas had few surviving RGCs. Injection of RGMa increased RGC survival after retinal ischemia. (**e**) Quantification of the density of surviving RGCs (±S.E.M.) at 14 days following ophthalmic artery ligation and treatments. RGMa significantly increased RGC survival (*P<0.001) after ischemia

To study the expression of RGMa and Neogenin in the adult rat retina, we performed colocalization studies with the RNA-binding protein RBMPS, a marker for RGCs. This showed that Neogenin and RGMa were both expressed by RGCs (Figure 1a). As Neogenin was expressed by RGCs, we assessed its involvement in cellular death after ischemia. To do so, we submitted whole-mount retinae to oxygen glucose deprivation (OGD, 1 h) followed by reperfusion. One day after insult, cell death was visualized using propidium iodide (PI) staining. Here, the relative PI intensity increased ~4-folds for OGD-treated retinae (*n*=6) when compared with controls (*n*=6; Figures 1a and b). Thus, OGD induced cellular death within the retina. Addition of RGMa (2 *μ*g/ml) directly to the medium just after OGD reduced PI intensity by ~45% compared with controls, indicating that RGMa promoted cell survival (Figures 1b and c). To determine whether this pro-survival effect was mediated by Neogenin, this receptor was neutralized using an antibody^12^ raised against the entire extracellular domain of Neogenin, which suppressed the pro-survival effect of RGMa, indicating that Neogenin mediates the RGMa effects (Figures 1b and c). Together, these results reveal that RGMa interaction with Neogenin promotes survival in an *in vitro* model for ischemic insult.

Next, we studied cell survival *in vivo* using a model of transient (30 min) ophthalmic artery ligation followed by assessment of RGC survival. Ligation of the ophthalmic vessels produces a uniform ischemic injury in the inner retina, the location of RGC cell bodies.^13^ RGMa was applied by intraocular injections (1 *μ*l at 1 *μ*g/*μ*l in PBS) at 3 and 10 days after ischemia, and the survival of Fluorogold pre-labeled RGCs was quantified at 14 days post-ischemia. In controls (*n*=5), the average density of RGCs was ~950 cells/mm^2^ (Figures 1d and e). Injection of RGMa significantly increased RGC densities by ~50% to ~1500 cells/mm^2^ (*n*=5; *P*<0.001; Figures 1d and e). Together with the above presented results, this revealed that the RGMa Neogenin pathway is involved in RGC death after ischemia.

### Generation of an antibody that alters Neogenin association with lipid rafts

**Figure 2:**
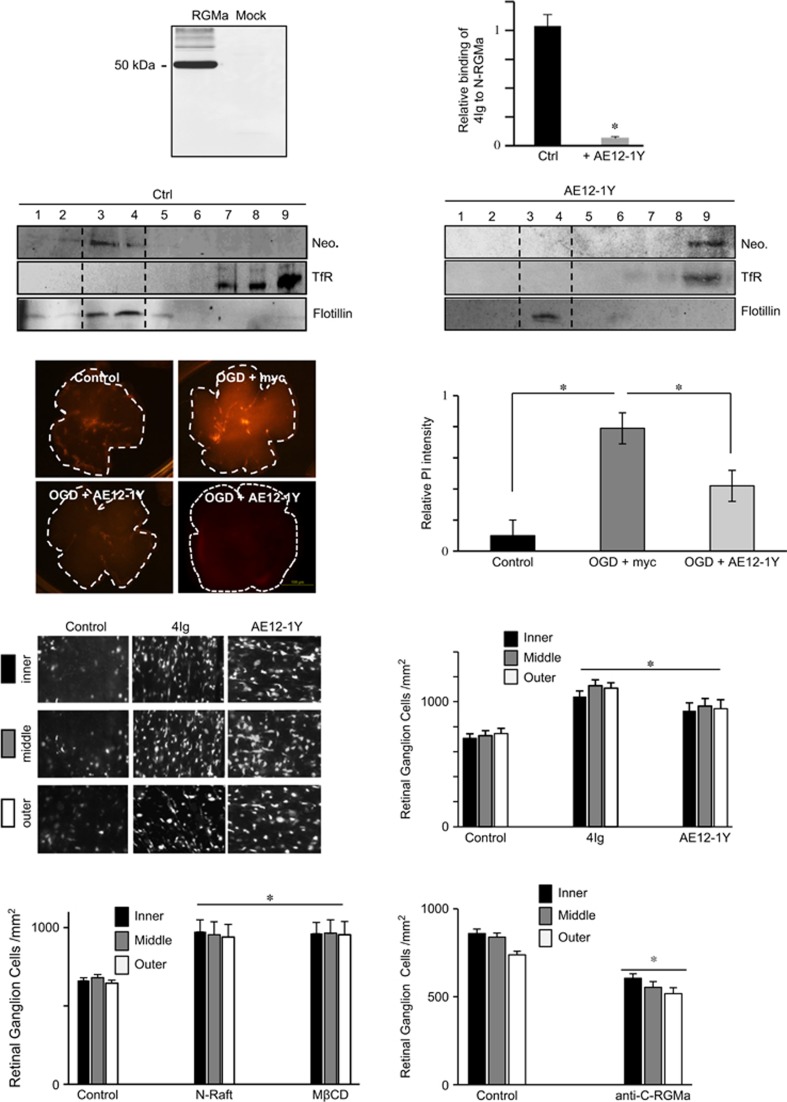
Mab AE12-1Y prevents Neogenin association with lipid rafts and promotes survival in the ischemic eye. (**a**) Western blot performed with AE12-1Y on RGMa or mock-transfected cells revealed that this antibody specifically recognized RGMa. (**b**) The interaction between the 4Ig domain of Neogenin and the N-Raft was assessed in an ELISA assay. AE12-1Y blocked the N-RGMa/Neogenin interaction, whereas a human IgG (control) did not. (**c** and **d**) Antibodies were injected into the E8 chick brain and 24 h after brains were collected and membrane fractionations prepared and analyzed in western blots. In controls, Neogenin localized with a marker for lipid rafts (Flotillin). Injection of mab AE12-1Y altered Neogenin localization, which colocalized with a marker for the heavy membrane fraction (transferin receptor, TfR). (**e** and **f**). Retinal whole mounts were submitted to OGD (1 h). One day after OGD, whole mounts were stained with PI to assess cellular death. (**e**) Representative micrographs 24 h following OGD. (**f**) Quantifications of experiments presented in **e** show that mab AE12-1Y significantly improved survival. (**g** and **h**) Panels taken at 14 days after ligation of the optic artery, show RGCs that were retrogradely labeled with Fluorogold. Control retinas, injected with human IgG at 3 and 10 days after ischemia had very few RGCs remaining at 14 days after axotomy; whereas, 4Ig and AE12-1Y injected retinas had many surviving RGCs. (**h**) Quantifications show that 4Ig and AE12-1Y significantly increased RGC survival following optic artery ligation. (**i**) Quantifications of experiments in which animals were treated with N-raft and M*β*CD following optic artery ligation show that these treatments significantly increased RGC survival compared with control peptide. (**j**) Quantifications of experiments in which anti-C-RGMa antibody was injected following optic artery ligation show that this peptide significantly promoted RGC death compared with control antibody. Values shown are mean±S.E.M. for six control retinas and eight 4Ig and eight AE12-1Y retinas in each experimental group. Significant differences were determined by ANOVA, followed by Tukey's test (*P<0.001)

Neogenin requires to be associated with lipid rafts to induce cell death.^14^ We have shown that recruitment toward lipid rafts involves a cis-interaction between the 4Ig domain in Neogenin and the N-Raft.^14^ Furthermore, overexpressing recombinant 4Ig to compete with endogenous domains displaced Neogenin from rafts toward non-raft fractions. To further validate this observation, and in order to develop therapeutics that promote CNS recovery, we generated human antibodies targeting RGMa by ProFusion mRNA display. Antibodies were selected on binding to RGMa and the ability to prevent the interaction between 4Ig and N-RGMa in an ELISA assay. AE12-1Y was generated as a high-affinity RGMa-selective mAb with comparable binding to human, rat and mouse RGMa. In western blots, AE12-1Y specifically detected a 55-kDa band in RGMa-transfected cells (Figure 2a). The antibody blocked the cis-interaction between the 4Ig domain in Neogenin and the N-Raft (Figure 2b). In contrast, a human IgG did not interfere in the interaction between these two domains. To confirm the hypothesis that Neogenin is recruited into lipid rafts by a specific interaction between 4Ig and N-Raft, we evaluated the effect of AE12-1Y on Neogenin association with lipid rafts. AE12-1Y was injected into the E8 optic tectum and the tecta were collected a day later for fractionation analyses (Figures 2c and d). In controls, full-length Neogenin localized exclusively to the raft fraction (anti-myc injection). As expected, AE12-1Y re-localized Neogenin from rafts to the heavy membrane fractions (Figures 2c and d), indicating that this antibody broke Neogenin association with lipid rafts.

### Blocking Neogenin association with lipid rafts promotes survival in models of retinal ischemia

The above-presented results suggest that the RGMa/Neogenin pathway is a key trigger of cellular death following ischemia. As we have shown that Neogenin requires association with lipid rafts in order to induce cellular death,^14^ we tested the role of Neogenin association with lipid rafts on retinal whole mounts following OGD. To do so, we first submitted retinal whole mounts to OGD in the presence of AE12-1Y (Figures 2e and f). In agreement with a role of Neogenin in stroke-induced death, the antibody significantly increased cell survival when compared with control (*n*=5; anti-myc). Relative PI intensity was reduced by 47% (4.2±1.4) in AE12-1Y-treated retinae (*n*=5) when compared with OGD+myc (*n*=5; 7.9±2.1).

Having shown that AE12-1Y promoted cell survival *in vitro*, we next studied whether blocking Neogenin association with lipid rafts can also have the same benefit *in vivo*. We performed ocular ischemia using ligation of the ophthalmic artery, and 4Ig and AE12-1Y (*n*=5 for each) were applied intraocularly (2 *μ*l at 1 *μ*g/*μ*l in PBS) at 3 and 10 days after ischemia (Figures 2g and h). Fourteen days post-ischemia, Fluorogold pre-labeled RGCs were quantified to evaluate survival. Similar to the injection of RGMa, the injection of both 4Ig and AE12-1Y increased RGC survival by ~50% (*P*<0.001; Figures 2e and h). To confirm that Neogenin localization in lipid rafts is involved in RGC death, we performed experiments with the N-raft peptide and methyl-beta-cyclodextrin (M*β*CD). The N-Raft peptide (2 *μ*l at 1 *μ*g/*μ*l in PBS) binds specifically to Neogenin to prevent its association with lipid rafts. M*β*CD (10 mg/kg) is a cholesterol chelator that disrupts lipid rafts. In agreement with above-presented results, we observed that N-Raft and M*β*CD treatments increased cell survival by ~50% (Figure 2i).

AE12-1Y has been raised against the N-Raft that regulates Neogenin association with lipid rafts. We have reported that the C-terminal part of RGMa interacts with Neognin to regulate various biological events. To ensure that the effect observed with AE12-1Y did not result from a neutralization of other aspects of RGMa function, we studied cell survival after ligation of the ophthalmic artery and injection of an anti-C-RGMa antibody (2 *μ*l at 1 *μ*g/*μ*l in PBS at 3 and 10 days after ischemia). Interestingly, C-RGMa antibody resulted in a decrease in RGC survival by ~30% (Figure 2j). This fits with the notion RGMa, acting in trans on Neogenin, may promote cell survival. It also suggests that the effect observed with AE12-1Y most likely resulted from its effect on Neogenin re-localization outside lipid rafts. Taken together, these results revealed that Neogenin requires to be associated with lipid rafts to induce RGC death after ischemia.

### Blocking Neogenin association with lipid rafts prevents caspase-3-mediated apoptosis following OGD

**Figure 3:**
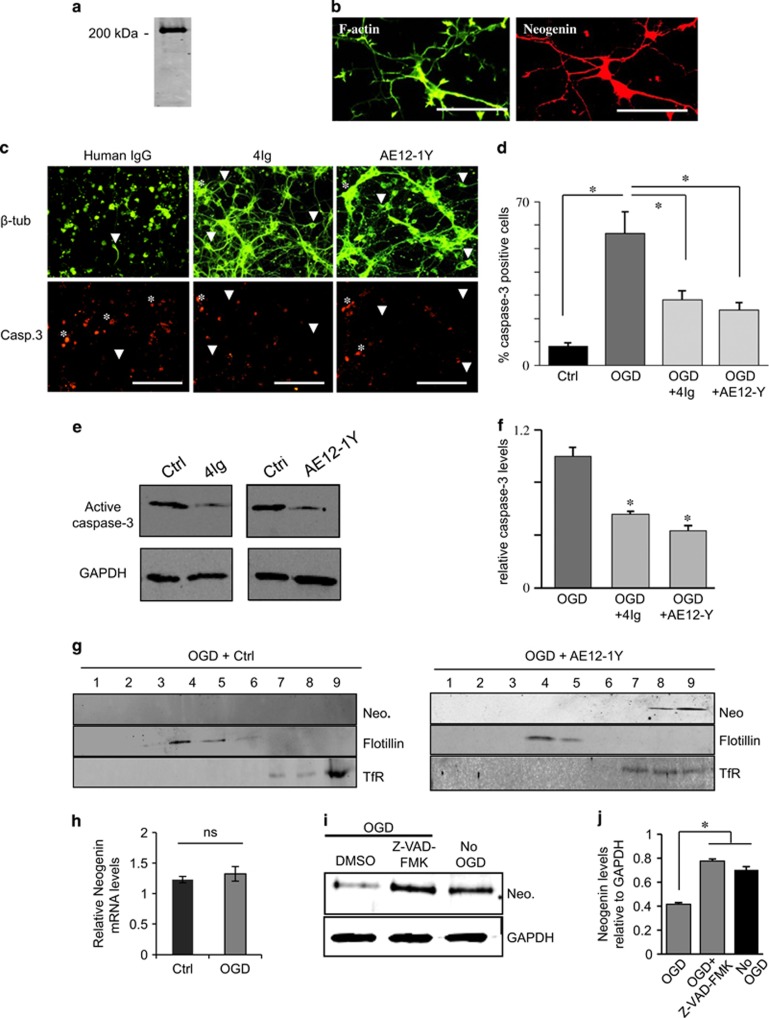
Blocking Neogenin association with lipid rafts decreases apoptosis and caspase-3 activation. (**a** and **b**) Neogenin is expressed in cortical neurons. Primary cortical neurons were cultured and submitted to (**a**) western blot for Neogenin and (**b**) double labeling for F-actin (Alexa-Fluor Phalloidin) and Neogenin. Western blot as well as immunofluorescence revealed a strong Neogenin expression. Bar, 100 *μ*m. (**c**) Primary cortical neurons were subjected to 1 h OGD and then treated with either human IgG (1 *μ*g/ml, control), 4Ig (1 *μ*g/ml) or AE12-1Y (1 *μ*g/ml) for 6 h. Cells were immune stained for active caspase-3 and βIII-tubulin. (**d**) Quantification of experiments presented in **c** revealed that OGD resulted in an increase of caspase-3-positive cells (*P<0.005). The addition of both 4Ig and AE12-1Y to the medium significantly decreased the number of caspase-3-positive cells (*P<0.005). Bar, 150 *μ*m. (**e**) Western blot analysis on cells following OGD plus treatment with control (human IgG), 4Ig and AE12-1Y. The intensity of active caspase-3 bands appeared weaker in the presence of 4Ig and AE12-1Y. (**f**) Quantification of western blot experiments showed that the presence of 4Ig and AE12-1Y significantly reduced caspase-3 activation (*P<0.005). (**g**) Cortical neurons were submitted to OGD in the presence of 1 *μ*g/ml of AE12-1Y or control (human IgG 1 *μ*g/ml) and fractionation was performed 1 h after ischemia. In control experiment, Neogenin (Neo.) signal could not be seen. In the presence of AE12-1Y, Neogenin colocalized with the heavy fraction marker transferrin receptor (TfR). Flotillin was used as a marker for lipid rafts. (**h**) Assessment of the relative Neogenin mRNA levels (relative to HPRT1) in OGD and control cultures revealed no significant (NS) difference. (**i**) Cortical neurons were treated with the pan caspase inhibitor Z-VAD-FMK (20 *μ*M) or DMSO (control) for 2 h and submitted to OGD for 30 min. Western blotting analysis was performed with Neogenin and GAPDH antibodies. In the presence of Z-VAD-FMK, levels for Neogenin appeared higher. (**j**) Neogenin levels relative to GAPDH were quantified, which demonstrates that incubation with Z-VAD-FMK restored Neogenin levels. Data from three independent experiments (*P<0.05)

In the developing CNS, Neogenin induces death by activating caspase-3, a known regulator of apoptosis. As caspase-3 is a key factor in cellular death following stroke, we decided to study whether or not preventing Neogenin association with lipid rafts has any influence on caspase-3 activation. Neogenin is expressed by cortical neurons (Figures 3a and b), thus we used these cultures as a model and performed OGD (1 h) in the presence of control antibody (human IgG, 1 *μ*g/ml), 4Ig (1 *μ*g/ml) or AE12-1Y (1 *μ*g/ml). Six hours after reperfusion, the morphology of control cells was markedly altered and neurons had lost more of their axonal/dendritic network (Figure 3c). In comparison, the presence of either 4Ig or AE12-1Y appeared to protect neuronal morphology. As expected, treatment with either 4Ig or AE12-1Y, also significantly reduced the number of caspase-3-positive cells following OGD, which was reduced by ~2-folds when compared with controls (28.1±3.9% for 4Ig and 23.6±3.2% for AE12-1Y *versus* 56.2±9.4 for the control). In western blots, addition of 4Ig and AE12-1Y reduced caspase-3 activation by ~2-folds, respectively, when compared with controls (human IgG; Figure 3f). The above-presented data suggest that Neogenin association with lipid rafts triggers caspase-3 activation and that application of either 4Ig or AE12-1Y can block this process. To further assess this possibility, we studied Neogenin raft localization in cortical neurons 1 h after OGD. As expected, when AE12-1Y was added to the medium, Neogenin was present in the heavy membrane fractions (Figure 3g). The situation appeared different when control antibody was added to the medium. In three independent experiments, we could not detect any Neogenin in fractions, suggesting that OGD led to Neogenin degradation. As we have shown that Neogenin requires to be cleaved by caspase-3 to be a pro-apoptotic molecule, we assessed the possibility that the absence of Neogenin following OGD resulted from proteolytical degradation by caspases. To do so, we first showed that OGD did not alter the mRNA levels for Neogenin in cortical neuron cultures (Figure 3h). Then, we studied the effect of the caspase inhibitor Z-VAD-FMK on Neogenin protein levels. Interestingly, pre-treatment with Z-VAD-FMK 2 h before OGD restored Neogenin levels to the one observed in control experiments (Figures 3i and j). This fits with our previous data showing that in order to activate caspase-3, Neogenin requires to be proteolytically processed by caspases.^3^ Taken together, these results suggest that Neogenin association with lipid rafts is involved in caspase-3 activation following ischemia.

### The 4Ig peptide reduced functional deficits and brain damage following Ischemia

**Figure 4:**
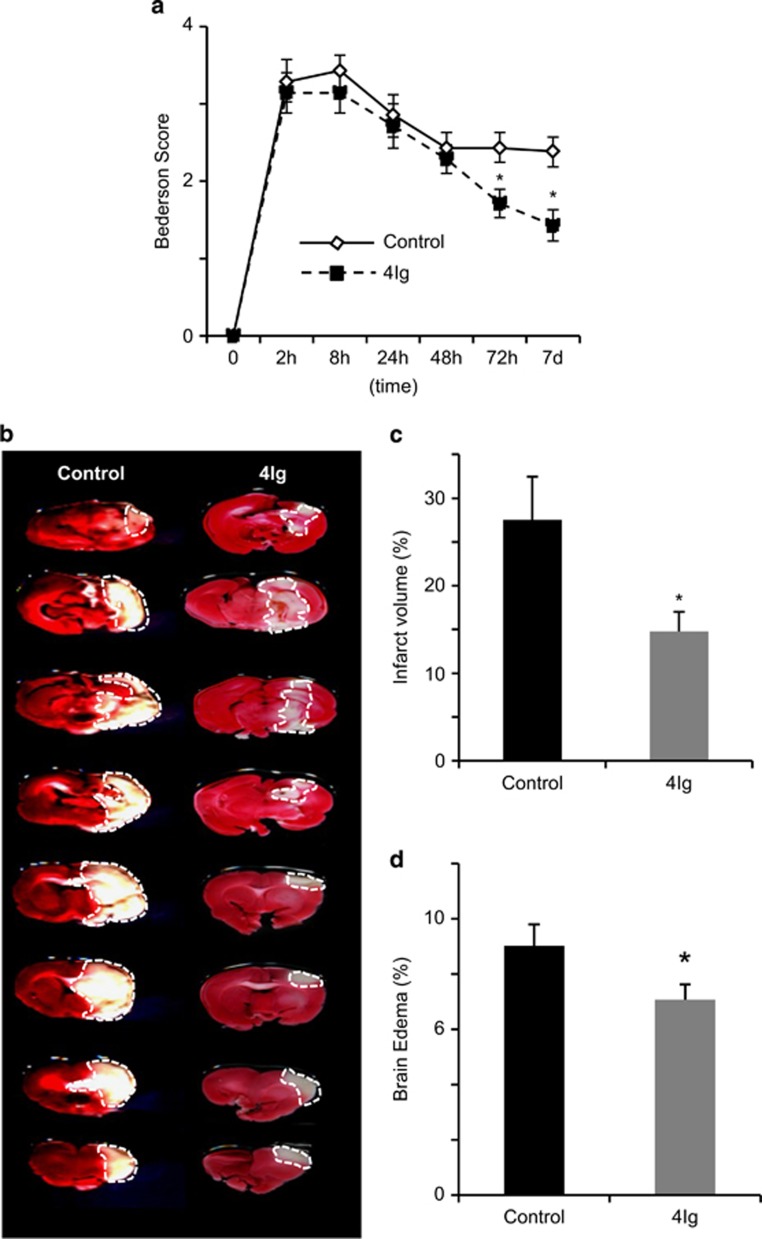
4Ig treatment reduces ischemic damage and promotes functional recovery after MCAO. Rats were subjected to MCAO followed by treatment with 4Ig (10 mg/kg, tail vein injection) or PBS (Control). (**a**) Behavioral assessment was performed over a 7-day period. This revealed that treatment with 4Ig led to a significant improvement of functional scores when compared with controls (*P<0.05). (**b**) Representative TTC-stained coronal brain sections (2 mm) from each group. (**c**) Infarct size was determined by TTC staining and expressed as the percentage of actual infarct volume in the total cerebral volume. Infarct size was significantly lower in 4Ig-treated rats when compared with controls. (*P<0.01). (**d**) Brain edema was determined by calculating the percentage of increase in ischemic *versus* healthy cerebral hemisphere. Treatment with 4Ig significantly reduced edema size (*P<0.05)

As preventing Neogenin association with lipid rafts proved to be neuroprotective following retinal ischemia, we tested the effects of this intervention on brain infarct development after transient MCAO (Figure 4). Cerebral ischemia can be experimentally induced by injecting either a heterogeneous or an autologous pre-formed clot into the MCA (thromboembolic stroke).^15, 16, 17^ Neurological scores were used to assess functional deficits and tail vein injection of 4Ig (10 mg/kg) was performed just after occlusion. Neurological scores were recorded before MCA occlusion and at 2 , 8 , 24 , 48 , 72 h and 7 days afterward (Figure 4a). At 2 and 8 h after MCA occlusion, all animals showed significant motor deficits, with median scores of ~3.2 for all groups (control, 4Ig; *n*=6 for each group). At 48 h after MCAO, neurological scores were not significantly different indicating that insults in the control and 4Ig groups were similar. Interestingly, 72 h following occlusion a significant difference was observed between controls and 4Ig animals. This difference increased slightly until termination of the experiments at 7 days following insult. Thus, 4Ig treatment produced a delayed behavioral improvement after stroke. Seizures were observed in three rats in the control group at 24 or 48 h after embolization. In contrast, no seizure activity was present in the 4Ig-treated cohort.

**Figure 5:**
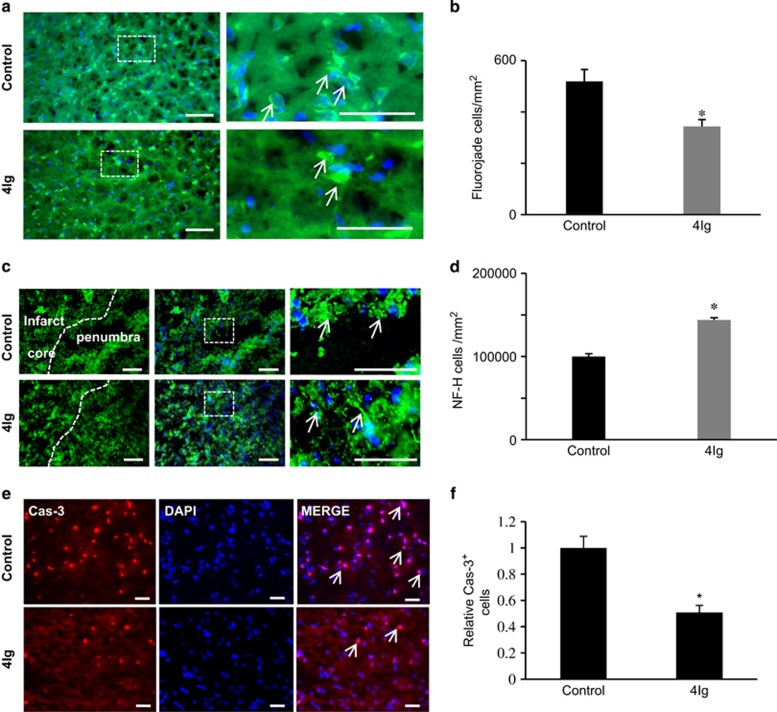
Treatment with 4Ig reduces neuronal damage after MCAO. Animals were submitted to MCAO and treated with PBS (control) or 4Ig (10 mg/kg, tail vein injection). One week after insult, brains were sectioned and neuronal damage was assessed. (**a**) Fluoro-Jade B labeling (green) of degenerating neurons revealed a higher number of damaged neurons in control *versus* 4Ig-treated animals. (**b**) Quantification indicated that the number of Fluoro-Jade-positive cells was significantly lower in 4Ig-treated brains when compared with controls (*P<0.01). (**c**) Fluorescence micrographs of peri-infarct area of the injured hemisphere. Columns (left to right) show NF-200 (NF-H) immunoreactivity with indications of the penumbra and core infarct, a merge of the preceding image with DAPI, and a higher magnification view of the inset white box in the merged images. Control samples showed sparse neurofilament immunoreactivity, whereas samples treated with the 4Ig peptide showed increased levels of NF-200 after MCAO. (**d**) Quantification of the relative mean number of NF-200-positive cells per mm^2^ (±S.E.M.) following MCAO. 4Ig significantly increased the relative level of NF-200 (*P<0.01), at 7 days after stroke. Scale bar, 100 *μ*m. (**e**) Representative pictures of control and 4IG treated cortical cultures following OGD and staining with DAPI or caspase-3. Bar, 100 *μ*m. (**f**) Quantification of experiments presented in **e** showed a significant reduction of caspase-3 activation following 4Ig treatment (*P<0.01)

To evaluate the effects of 4Ig treatment on brain damage, we performed a TTC staining and compared the relative volumes of brain tissue between the infarcted and non-infarcted hemispheres of the brain. Brain infarct reduced by~50% following intravenous injection of 4Ig when compared with control: brain infarct volume in control and 4Ig groups was 27.5±4.9% and 14.7±2.2%, respectively, at 7 days after MCAO (*P*<0.005; Figure 4c). Similarly, the size of the edema was significantly reduced and was of 7.0±0.5% in the 4Ig-treated group and 9.0±0.7% in the control group (*P*<0.05; Figure 4d). These data taken together with the *in vitro* data suggest that 4Ig treatment preserved neurons following ischemic insult. To confirm that 4Ig prevented neuronal death, we performed a Fluoro-Jade B staining, which reveals dying neurons.^18^ Brain sections were stained and the number of Fluoro-jade-positive cells quantified. Treatment with 4Ig reduced the number of positive neurons by ~35% when compared with control (519.4±46.1 and 344.1±25, respectively; Figure 5b).

To assess neuronal integrity after stroke and 4Ig treatment, NF-200 immunostaining^19^ was examined in the peri-infarct region of the injured cerebral hemisphere (Figure 5c). The relative mean number of NF-200-positive neurons was significantly increased after 4Ig administration (143,225±/mm^2^) compared with the control group (97,829±/mm^2^) (*P*<0.01; Figure 5d), at 7 days after ischemia.

Finally, to determine whether 4Ig prevents neuronal death by blocking caspase-3 activation, we performed an anti-caspase-3 staining in the peri-infarct region 7 days after ischemia. Together these results show that treatment with the 4Ig domain of Neogenin prevents neuronal damage following stroke, which results in improved functional recovery.

### AE12-1Y reduced functional deficits and brain damage following ischemia

**Figure 6:**
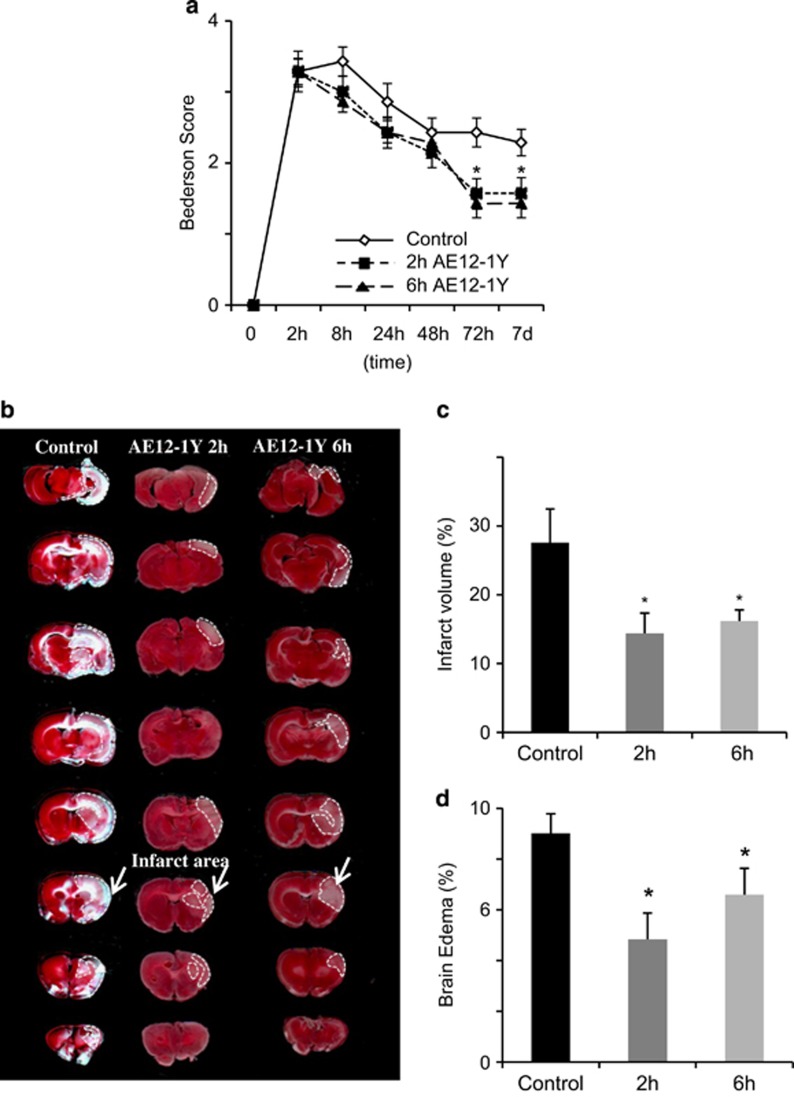
Delayed treatment with AE12-1Y reduces ischemic damage and promotes functional recovery after MCAO. Rats were subjected to MCAO followed by delayed treatment (2  and 6 h after MCAO) with AE12-1Y (10 mg/kg, tail vein) or human IgG (10 mg/kg, tail vein; control). (**a**) Behavioral assessment was performed using the Bederson Score over a 7- day period. Treatment with AE12-1Y (delayed 2  and 6 h) led to a significant improvement of functional scores when compared with controls (*P<0.05). (**b**) Representative TTC-stained coronal brain sections (2 mm) from each group. (**c**) Infarct size was determined by TTC staining and expressed as the percentage of actual infarct volume in the total cerebral volume. Infarct size was significantly lower in AE12-1Y-treated (2 and 6 h) rats when compared with controls (*P<0.01). (**d**) Brain edema was determined by calculating the percentage of increase in ischemic *versus* healthy cerebral hemisphere. Treatment with AE12-1Y significantly reduced edema in both (2 and 6 h) experimental paradigms

As 4Ig reduced functional deficits following MCAO, we decided to confirm the role of Neogenin association with lipid rafts using the AE12-1Y antibody. In order to evaluate whether delayed application of AE12-1Y can also reduce stroke-induced damages, we injected the antibody 2 and 6 h after MCAO (*n*=6 for each group). Neurological scores were recorded before MCA occlusion and at 8 , 24 , 48 , 72 h and 7 days afterward (Figure 6a). When the treatment was initiated 2 h after ischemia, the initial neurological scores were not significantly different in both the control (human IgG antibody) and AE12-1Y-treated animals. Similar to 4Ig treatment, the 2 h delayed application of AE12-1Y resulted in a significant improvement of behavioral scores 72 h following occlusion (Figure 6a). This difference was maintained until termination of the experiments at 7 days following ischemic insult. The same trend was observed when treatment with AE12-1Y was initiated 6 h following ischemia. Thus, not only treatment with AE12-1Y resulted in behavioral improvement after stroke but it could be delayed by 6 h with the same behavioral benefit. Seizures were observed in three rats in the control group at 24 or 48 h after embolization. In contrast, no seizure activity was present in the AE12-1Y-treated cohort.

To study the effects of AE12-1Y treatment on brain damage, we performed a TTC staining, which revealed a ~50% reduction of brain infarct following intravenous injection of AE12-1Y at 2 h, when compared with controls (Figures 6b and c). A similar reduction (~ 45%) was observed when treatment started 6 h after insult (Figures 6b and c). Edema size was decreased by ~45% when treatment was performed 2 h following MCAO and was 4.8±0.5% in the AE12-1Y-treated group and 9.1±0.8% in the control group (*P*<0.05). Reduction of the edema size was less pronounced (~35%) when treatment was performed 6 h following stroke, nevertheless a significant difference was still observed (*P*<0.05; Figure 6d).

**Figure 7:**
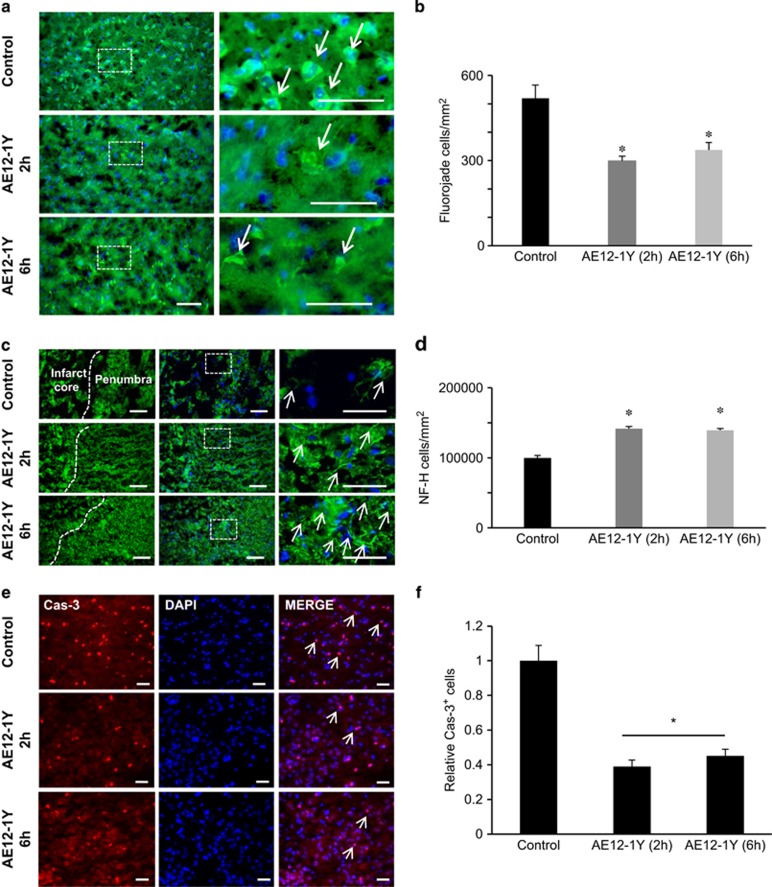
Treatment with AE12-1Y reduces neuronal damage after MCAO. Animals were submitted to MCAO and treated with human IgG (control) or AE12-1Y at 2 or 6 h following ischemia. One week after insult, brains were sectioned and neuronal damage was assessed. (**a**) Fluoro-Jade B labeling (green) of degenerating neurons revealed a higher number of damaged neurons in control *versus* AE12-1Y-treated animals. (**b**) Quantification indicated that the number of Fluoro-Jade-positive cells was significantly lower in AE12-1Y-treated (2 and 6 h) brains when compared with controls. (**c**) Fluorescence micrographs of peri-infarct area of the injured hemisphere. Columns (left to right) show NF-200 (NF-H) immunoreactivity with indications of the penumbra and core infarct, a merge of the preceding image with DAPI and a higher magnification view of the inset white box in the merged images. Control samples showed sparse neurofilament immunoreactivity, whereas samples treated with AE12-1Y showed increased levels of NF-200 after MCAO. (**d**) Quantification of the relative mean number of NF-200-positive cells per mm^2^ (±S.E.M.) following MCAO. AE12-1Y significantly increased the relative level of NF-200 (*P<0.01), at 7 days after stroke. Scale bar, 100 *μ*m. (**e**) Representative pictures of control and antibody AE12-1Y treated cortical cultures 2 or 6 h following OGD and staining with DAPI or caspase-3. Bar, 100 *μ*m. (**f**) Quantification of experiments presented in **e** showed a significant reduction of caspase-3 activation following AE12-1Y treatment (*P<0.01)

To further demonstrate that AE12-1Y prevented neuronal death, we performed a Fluoro-jade B staining (Figure 7a). Treatment with AE12-1Y, 2 h following stroke, reduced the number of positive neurons by ~45% when compared with controls: number of Fluoro-jade cells in AE12-1Y and control animals was 519±46.3 and 298.7±10.8, respectively (Figure 7b). Similarly, injection of AE12-1Y 6 h after MCAO reduced of Fluoro-jade cells (337.6±25.9) by ~40% when compared with controls. To assess neuronal integrity, NF-200 immunostaining was examined in the peri-infarct region of the injured cerebral hemisphere 7 days after ischemia (Figure 7c). The relative mean number of NF-200-positive neurons was significantly increased when AE12-1Y was injected 2 h after MCAO (141 911±2956/mm^2^) compared with the control group (97 829±2356/mm^2^) (*P*<0.01; Figure 7d). Injection 6 h after ischemia also significantly increased the number of NF-200-positive cells (139 469±2428/mm^2^). Together these data reveal that (i) blocking Neogenin association with lipid rafts prevents neuronal damages following ischemia and that (ii) this treatment can be delayed and still results in significant functional benefits.

## Discussion

This study contributes to our understanding of the mechanisms that control neuronal cell death following stroke, and reports several key findings. (i) Soluble recombinant RGMa significantly increased neuronal survival following stroke. (ii) The pathway through which RGMa mediates survival following ischemia involves Neogenin. (iii) Neogenin pro-death activity following stroke involves both an association with lipid rafts and activation of caspase-3. (iv) Blocking Neogenin association with lipid rafts restores brain functions even when antibody treatment was performed 6 h following stroke. These data provide the first evidence that receptor association with lipid rafts can be targeted to promote cell survival following stroke.

We have previously shown that RGMa promotes cell survival through association with Neogenin in the developing brain.^3^ Based on this work, we hypothesized that RGMa may interact with Neogenin to promote neuronal survival following ischemia. Our data demonstrate that RGMa promotes cell survival in retinal whole mounts following OGD and that Neogenin mediates this effect. Interestingly, Netrin, another Neogenin ligand has also been shown to promote neuronal survival following stroke.^20, 21^ Although still elusive, the mechanism by which Netrin-1 promotes survival following stroke, appears different than the one uncovered in this study for two reasons. First, location of Neogenin-positive cells differed from that of netrin-1-positive cells.^9^ Second, administration of Netrin-1 protected infarct tissue from p53-mediated apoptosis via interaction with DCC,^20^ another Netrin receptor with 88% homology to Neogenin. Thus, our study uncovers a new mechanism by which a dependence receptor protects neurons from death following stroke.

Our study also demonstrates that Neogenin requires to be associated with lipid rafts to induce neuronal death following stroke. Cholesterol, the main component of lipid rafts, has been the subject of numerous studies following stroke.^22, 23^ For instance, cholesterol synthesis inhibition by means of statins is known to be neuroprotective following stroke.^23, 24^ Although statins clearly promote survival, the mechanism of their neuroprotective effects is only partly understood and is likely to reflect an action on several pathways. Indeed, treatment with simvastatin has been shown to reduce NMDA receptors (NMDAR1) in lipid rafts, thereby protecting neurons from NMDA-induced neuronal death.^25^ Another protective mechanism elicited by statins is due to the inhibited synthesis of isoprenoid intermediates, which serve as lipid attachments for a variety of intracellular signaling molecules such as nitric oxide.^26^ Our study adds another possible mechanism that accounts for the benefit of statins following stroke. Indeed, by preventing Neogenin association with lipid rafts statins may well prevent the unwanted effect of this pathway.

Current therapeutic procedures for stroke have focused on (i) using tissue plasminogen activator and (ii) inhibiting excitotoxicity. However, both treatments have very narrow therapeutic windows^1, 27^ and targeting molecular pathways involved in the subacute phase of stroke may provide a larger therapeutic window. Neuronal cells mostly die by apoptosis during ischemic brain injury.^28^ Thus, targeting signaling pathways involved in apoptosis may ensure neuroprotective efficacy over a larger time window. Caspase-3, a key apoptotic factor, has been shown to be upregulated both in experimental Imodels and human brain following stroke.^29^ Pharmacological inhibition of this enzyme has been found to be neuroprotective following insult.^28^ Caspase activation occurred up to 9 h after MCA occlusion, and that the ischemic damage could be reduced by caspase inhibitors injected up to 9 h after reperfusion. Thus, targeting pathways that lead to caspase activation may extend the treatment window following stroke. As Neogenin activates caspase-3 to induce apoptosis, we tested whether it can promote neuronal survival over an extended time window. Interestingly, neuronal survival and function's recovery were improved even when treatment was applied 6 h following MCAO. Hence, targeting Neogenin may offer a novel therapeutic procedure for the treatment of stroke.

## Materials and Methods

### Antibody generation and affinity measurements

AE12-1Y was derived from AE12-1, a fully human anti-RGMa mAb generated via PROfusion mRNA display technology. AE12-1Y is a high-affinity, RGMa-selective mAb. It exhibits comparable binding affinity to human, rat and mouse RGMa, and neutralizes RGMa activity in functional assays (chemotaxis, neurite outgrowth and N-RGMa-4Ig interaction assay).

### Protein injections and lipid raft fractionations

AE12-1Y antibody (1 mg/ml; 5 *μ*l) was injected in chick E8 optic tectum and tecta were collected 24 h later. Tecta (three tecta for each set of experiment) and/or cells were solubilized in chilled buffer (25 mM Tris-HCl, pH 7.4, 150 mM NaCl, 5 mM EDTA and protease inhibitor cocktail) and passed through G25 and G30 needles, respectively, and centrifuged at 800 *g* for 10 min at 4 °C. Cold Triton X-100 was added to the post nuclear supernatant to a final 1% and incubated on ice for 1 h. In all, 2X volume of 2 M sucrose was added and sample placed at the bottom of a sucrose density gradient (0.9–0.8–0.75–0.7–0.6–0.5–0.4–0.2 M) and centrifuged at 38 000 r.p.m. for 16 h in SW 60 rotor (Beckman Instruments Inc., Mississauga, ON, Canada). In total, 400 μl fractions were collected from top (top numbered 1 and next eight fractions numbered consequently). The cell preparations were run on western blots and probed with Neogenin (H-175; Santa Cruz, Dallas, TX, USA), Flotillin (Santa Cruz) and TfR antibody (abm) for 2 h at RT. Odyssey goat anti-mouse secondary antibody (1 : 4000; LI-COR, Lincoln, NE, USA) was used for 1 h at RT.

### Injections and medications

In order to evaluate the effects of 4Ig and AE12-1Y, on RGC survival and regeneration after ligation of the optic artery, we delivered 2 *μ*l intraocular injection of RGMa (500 ng/*μ*l), 4Ig (500 ng/*μ*l) and AE12-1Y (500 ng/*μ*l) solutions at 3 and 10 days after injury. Retinal ischemia was carried out as previously described.^30, 31^ One week before ischemia, animals received stereotaxic injections of 2% Fluorogold into the superior colliculus, the brain target of RGCs, in order to retrogradely pre-label all RGCs in the retina for future quantification.

Intraocular injections was described previously.^31^ Briefly, rats were anesthetized with 2% isoflurane in a mix of O_2_ and then the cornea was anesthetized using Alcaine eye drops (Alcon) before intraocular injections. A pulled glass micropipette attached to a 10 *μ*l Hamilton syringe via a hydraulic coupling through PEEK tubing was used to deliver 4Ig, and antibody solutions into the vitreous of the eye, posterior to the limbus.^32^ The pipette was held in place for 5 s after injection and slowly withdrawn from the eye to prevent reflux. Following injection, the cornea was covered with ophthalmic ointment to prevent desiccation and animals were returned to their normal housing.

Animals were killed at 14 days after optic artery ligation, the eyes were enucleated, the cornea and lens were removed, and the remaining eye cups containing the retinas were fixed in 4% paraformaldehyde for 1 h. The retinas were then removed, flat-mounted and coverslipped using 50  :  50 glycerol/ PBS. Fluorogold staining in RGCs was visualized using an Andor iXon 885+ electron-multiplying charge-coupled device camera attached to a Leica DM LFSA microscope (Concord, ON, Canada). Moreover, Sutter Lambda XL (Quorum Technologies, Guelph, Canada) with a liquid light guide was used as a source of illumination. The densities of RGC were measured at the inner (1/6 retinal eccentricity), midperiphery (1/2 retinal eccentricity) or outer retina (5/6 retinal eccentricity) of each quadrant of the flat-mount (defined distances from the center of the retina). This cell count was divided by a factor of 0.08, to yield an extrapolated RGC density per unit area of cells/mm^2^.^33^

Rats in the M*β*CD group received one intraperitoneal injection of M*β*CD at 1000 mg/kg immediately following optic artery ligation and then daily i.p. injections until killing. Control rats received equivalent volumes as described above. The rats were housed singly in a temperature-controlled room at 24 °C with a 12-h light–dark cycle. Water and food were provided *ad libitum*.

### *In vitro* stroke

#### Retinal whole-mount cultures

Sprague–Dawley rat pups of either sex (P14; Charles River (Montreal, QC, Canada)) were anesthetized using 4% isoflurane and decapitated. After taking eyeballs, they were briefly washed with PBS and 10% germicide and placed in cold medium. Retinas were flat mounted with the ganglion cell layer facing upward on Millicell-CM chamber (0.4 *μ*m culture plate insert, 30mm diameter; Millipore,Bedford, MA, USA). The retinas were cultured for 2 days (37 °C, 5% CO_2_) in 1 ml of culture medium, which contained Neurobasal medium, 10% fetal bovine serum, 2% B-27 supplement, 1% glutamine and 3% antibiotic/antimycotic (all from Invitrogen, Burlington, ON, Canada).

#### Cortical neuron cultures

Mouse E16 cortical neurons were prepared as we described earlier.^34^ Briefly, cortices were dissected and dissociated in 0.05% Trypsin, 0.04% EDTA–PBS solution at 37 °C for 20 min. Cells were centrifuged at 500 r.p.m. for 5 min and seeded in B-27 supplemented Neurobasal medium (Life Technologies, Burlington, ON, Canada) at 100 000 cells per well of 12-well poly-L-Lysine-coated plates (Greiner, Monroe, NC, USA).

Retinal whole-mount and cortical neurons cultures were subjected to OGD injury as described previously.^35^ Briefly, cultures were washed twice in glucose-free Earl's balanced salt solution (EBSS: 116.4 mM NaCl, 0.8 mM MgSO_4_, 5.4 mM KCl, 2.6 mM NaH_2_PO_4_, 26.2 mM NaHCO_3_, 1.8 mM CaCl_2_ and 20.1 mM HEPES pH 7.4), and incubated in oxygen- and glucose-free EBSS in a humidified anaerobic culture incubator filled with 93% N_2_, 5% CO_2_ and <2% O_2_ for 1 h. After OGD, the cultures were replaced into neurobasal medium plus RGMa, RGMa antibody, 4Ig, PBS (control) or control antibody (human IgG). Cells were maintained in a normal CO_2_ incubator until fixation with 4% PFA or collection for membrane preparations.

To assess cell survival in whole mounts, PI (final concentration, 2 g/ml) was added directly to the medium and images were taken at the same intensity using a fluorescence microscope every 24 h after explantation. Images of PI-stained retinas were analyzed using ImageJ, and the fluorescence intensity at each time point was quantified, and background fluorescence was subtracted.

To study apoptosis in cortical neurons, neurons were fixed in 4% PFA and permeabilized with 0.1% Triton X100 and co-labeled with anti-caspase-3 (1  :  750; Cell Signaling), and *β*III-tubulin (Covance, Cederlane, Burlington, ON, Canada) at 4 °C. Next day, cells were washed and incubated with Alexa 488 anti-mouse and Alexa 555 anti-rabbit secondary antibodies (1  :  500; Molecular Probes) along with DAPI/PBS for 1 h at RT.

For western blots, neurons were lyzed in RIPA buffer and loaded on a 10% acrylamide gel before transfer onto a nitro-cellulose membrane. Blots were probed with (i) an anti-caspase-3 antibody and (ii) an anti GAPDH antibody for loading control.

For treatment with pan-caspase inhibitor (Z-VAD-FMK), cortical neurons were pretreated with the inhibitor (20 *μ*M) or DMSO for 2 h and then subjected to OGD for 30 min. The cultures were lysed and probed with anti-Neogenin antibody after western blotting. The pixel areas for each band were quantified for identical areas and the relative amounts to GAPDH levels were quantified.

### Cerebral ischemic model and assessment

For thromboembolic ischemia, a preformed blot clot was injected into the MCA via the internal carotid artery.^17, 36^ Sprague–Dawley rats weighing 200–250 g were anesthetized with 3.0% isoflurane and then maintained with 1.5% isoflurane in a mixture of 30 : 70 O_2_ and NO_2_ with a face mask during surgery. Body temperature was maintained at 37 °C with a heating pad for the duration of surgery and the immediate postoperative period until animals recovered fully from anesthesia. A 1.5-cm longitudinal incision was made in the midline of the ventral cervical skin. The right common carotid artery, right internal carotid artery and right external carotid artery (ECA) were exposed. The distal portion of the ECA was ligated and cut. A modified polyethylene-10 catheter, filled with bovine thrombin (Thrombostat, TM Warner-Lambert Co., Morris Plains, NJ, USA), was introduced into the lumen of the right ECA via a small puncture. Ten microliters of blood were withdrawn into the catheter and retained for 15 min to allow formation of a clot. Once the clot was formed, the catheter was advanced 17 mm into the internal carotid artery until its tip was 1-2 mm away from the origin of the MCA. The preformed clot in the catheter was then injected, and the catheter removed. After surgery, the wound was closed, and the animal was returned to its cage.

#### Experimental design

Animals were randomly assigned into each group (*n* =6). Animals of each group received PBS, human IgG (control, 10 mg/kg), 4Ig (10 mg/kg) and mab AE12-1Y (10 mg/kg). Treatments were injected i.v.via tail vein, starting immediately or every 2 and 6 h after embolization.

### Infarct volume and edema size

Quantification of infarct volume and edema was performed as previously described.^17, 36^ The infarct volume was expressed as a percentage of the total volume from the ipsilateral hemisphere. Brain edema was determined by calculating the volume difference between the two hemispheres and dividing by the volume of the left hemisphere. Seven days after MCA occlusion, the anesthetized rats were killed. The brains were removed from the skull and cooled in ice-cold saline for ~5 min. For morphometric examination, 2-mm-thick coronal sections were cut using a rat brain matrix. A total of eight coronal sections were collected, and the sections were stained using a 2% 2, 3, 5-triphenyltetrazolium chloride solution. The infarct appeared pale white on a background of red normal brain. The stained brain sections were scanned with the Scan Jet (Hewlett Packard, Palo Alto, CA, USA) flatbed scanner. Determinations of infarct volume and edema in six groups were blinded. The infarct volume was calculated using the following formula: infarct volume=(left hemisphere volume–(the right hemisphere volume–measured infarct volume))/left hemisphere volume. Brain swelling was determined using the following formula: swelling (edema)=(right hemisphere volume–left hemisphere volume)/left hemisphere volume. The infarct volume and brain swelling were expressed as percentages.

### Neurological deficits and seizures

Neurological deficits and seizure activity of each rat were evaluated at 2, 8, 24, 48, 72 h and 1 week after ischemic injury by an observer who had no knowledge of which procedure had been performed. Neurological deficits and seizure activities were classified with Bederson's and Racine's scoring systems.^37, 38^ Seizure activity was classified with the Racine scale. Mortality was also recorded. Bederson's scoring system: 0, no observable deficit (normal); 1, forelimb flexion (moderate deficits) ;2, forelimb flexion plus decreased resistance to lateral push (moderate deficits); 3, unidirectional circling (severe deficits); 4, unidirectional circling plus decreased level of consciousness (severe deficits).

### Quantitative real-time PCR

To quantify Neogenin transcript levels following OGD in mouse cortical neurons, total RNA was isolated using a RNeasy Mini Kit (Qiagen, Toronto, ON, Canada) as described by the manufacturer. Purified total RNA (500 ng) was reverse transcribed into cDNA using Superscript II First Strand Synthesis System (Invitrogen). Pre-designed Taqman probes (Applied Biosystems-Life Technologies, Mississauga, ON, Canada) were used to assess mRNA expression: Neogenin (Mm00476326_m1) and the reference transcript HPRT1 (Mm01545399_m1). To verify specific amplification of our target gene, negative controls (cDNA without primers and primers without cDNA) were used. The PCR amplification was carried out in a 384-well plate using Taqman Master Mix (Applied Biosystems, Mississauga, ON, Canada) using a final concentration of 10ng of cDNA generated from total isolated RNA on a 7900HT Fast Real-Time PCR System (Applied Biosystems) with the following conditions: an enzyme activation step at 50 °C for 2 min and a 95 °C denaturation step for 10 min, followed by 40 cycles at 95 °C for 15 s, 60 °C annealing for 1 min and a 72 °C extension for 30 s. The data were analyzed using the Applied Biosystems Sequence Detection System (SDS) software 2.2. The threshold cycle (C_t_) values were averaged from three independent experiments and were normalized to HPRT1 levels and represented as 2^−ΔCt^ (ΔC_t_= C_t(Neogenin)_- C_t(HPRT1)_).

## Abbreviations

RGMa: repulsive guidance molecule a
OGD: oxygen glucose deprivation
MCAO: middle cerebral artery occlusion
RGC: retinal ganglion cells
CNS: central nervous system
N-Raft: N-terminal portion of RGMa
4Ig: Neogenin immunoglobulin domain
M*β*CD: methyl-beta-cyclodextrin
mAb: monoclonal antibody
